# Neonatal T cells unleash innate powers to combat congenital cytomegalovirus infection

**DOI:** 10.1172/JCI187789

**Published:** 2025-01-02

**Authors:** Simon Grassmann

**Affiliations:** Immunology Program, Memorial Sloan Kettering Cancer Center, New York, New York, USA.

## Abstract

Approximately 1 in 200 newborns worldwide are affected by congenital cytomegalovirus (CMV). Most of these cases are asymptomatic due to successful control of the infection by the newborn’s immune system. In this issue of the *JCI*, Semmes et al. characterized the cellular immune response in cord blood of neonates with CMV infection. The authors found that conventional T cells with NK-like features expanded during congenital CMV infection. To exert their antiviral function, these cells relied on Fc receptors, recognizing virus-infected cells bound by IgG. Thereby, the fetal and maternal immune system can optimally cooperate to control CMV infection: maternal IgG crossing the placenta opsonizes virus-infected cells subsequently lysed by neonatal NK-like T cells. This finding suggests that innate-like programming of conventional T cells may have evolved to combat congenital CMV infection, offering insights that could inform the development of future therapies.

## Congenital CMV infection remains a threat to newborns

Approximately 1 in 200 newborns are born with congenital cytomegalovirus (CMV) infection (1, 2), treatment of which is difficult. To date, the only available option for neonates is the use of antivirals. During pregnancy, antivirals and hyperimmune globulin can reduce the rate of transmission. However, after transmission occurs, such treatments do not prevent CMV-dependent complications (3, 4). In addition, preexisting immunity of the mother does not always confer complete protection: although fetuses of CMV-seropositive mothers are protected from CMV infection, a subset can still be congenitally infected and develop complications, including hearing loss (5).

A possible angle to develop new therapeutic concepts is to focus on the congenital infections that are successfully controlled. Despite the high prevalence of congenital CMV infection, only 10%–20% of newborns show clinical signs and even fewer are impacted by CMV-related complications such as neurodevelopmental delays or sensorineural hearing loss (1, 6). This finding suggests that in many neonates congenital CMV infection is successfully controlled by the maternal and neonatal immune system. Understanding the mechanisms behind this protection could lead to the development of more successful therapies.

## Fetomaternal cooperation for control of CMV infection

Responses against herpesviruses like CMV, aside from those related to the adaptive immune system, include several mechanisms of the innate immune system that are specifically tailored to combat viral antigens. Different strains of CMV have coevolved with their respective mammalian hosts for millions of years (7). This coevolution led to host specificity (murine CMV [MCMV] in mice, human cytomegalovirus [HCMV] in humans) as well as immune evasion strategies for the pathogen (8). However, the evolutionary pressure also led to an adaptation of the host — especially in the innate immune system. NK cells — the canonical antiviral cells of the innate immune system — are a crucial example. In mice, NK cells evolved to directly recognize m157, a viral antigen encoded by MCMV, via the activating receptor Ly49H (9, 10). In humans, the activating receptor NKG2C recognizes CMV-encoded peptides presented by human leukocyte antigen-E (HLA-E) (11).

Innate mechanisms for viral control give the host a unique advantage. While cells of the adaptive immune system need several days to expand, NK cells can exert their antiviral function immediately. This mechanism is likely even more important for newborns: the adaptive immune system needs time to develop, and fetuses are thought to have a relatively immature adaptive immune system (12). Moreover, adaptations during pregnancy that allow for maternofetal tolerance may further inhibit the fetus’s adaptive immune system (13).

The relatively weak adaptive immune system of the fetus is counteracted by an additional mechanism: maternal antibodies can cross the placenta and play a crucial role for fetus protection (14). For this function, the innate immune system of the fetus is again important: via Fc receptors, NK cells can use the maternal IgG to recognize virus-infected cells (15). Together with activating receptors such as NKG2C, NK cells can then identify and lyse virus-infected target cells.

## Innate reprogramming of T cells in congenital CMV infection

**Figure 1:**
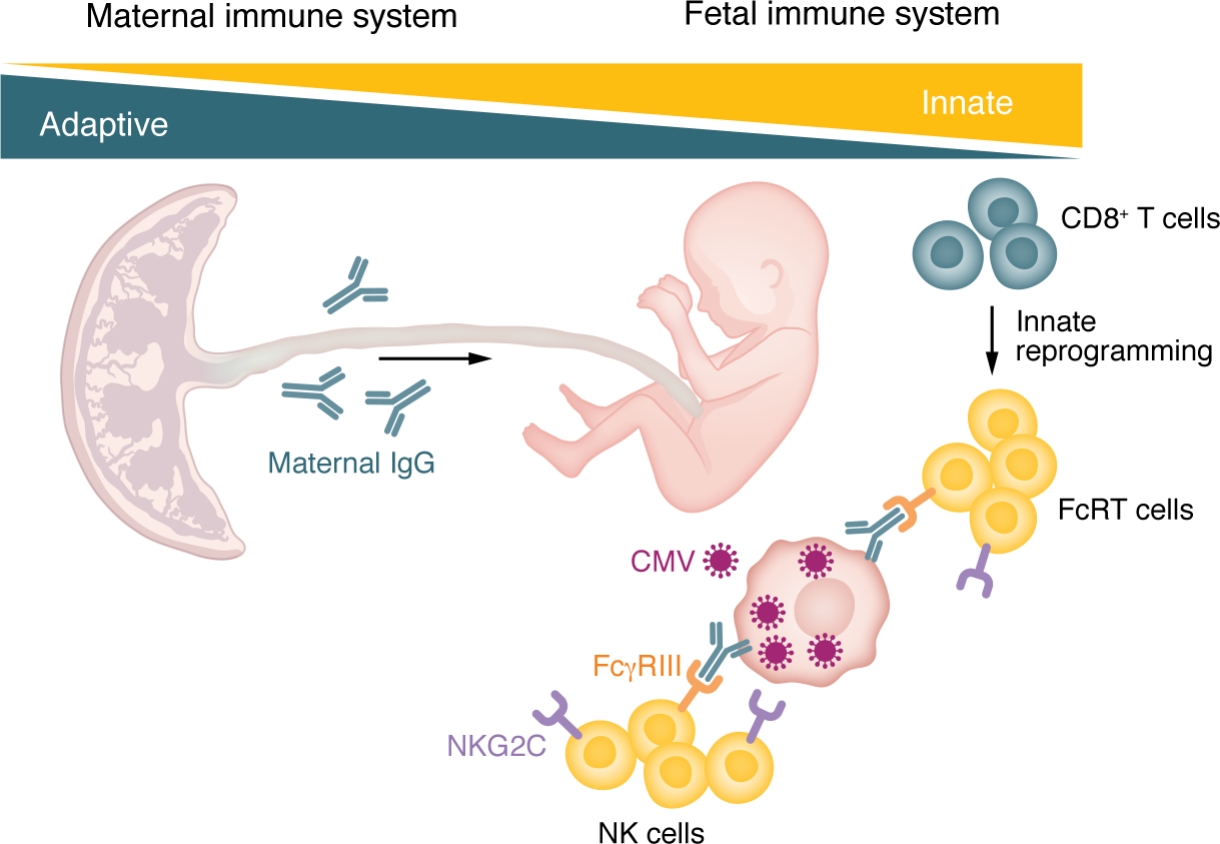
Fetomaternal immune systems collaborate in the control of congenital CMV infection. During congenital CMV infection, maternal IgG against CMV crosses the placenta. Conventional CD8^+^ T cells upregulate FcγRIII and NKG2C, transdifferentiating into FcRT cells. Maternal IgGs elicit antibody-dependent cellular cytotoxicity (ADCC) by FcRT cells and NK cells. NK cells also target and lyse virus-infected cells directly via NKG2C-activating receptors.

In this issue of the *JCI*, Semmes et al. found that not only NK cells but also conventional CD8^+^ T cells participated in this fetomaternal collaboration (16). During congenital CMV infection, conventional CD8^+^ T cells upregulated NK cell markers, including Fc receptors (specifically, Fcγ receptor III: FcγRIII) and NKG2C (Figure 1). Such NK-like T cells (also called FcRT cells) have been observed in adult virus infection (17, 18), but the role of these cells in congenital infection is incompletely understood.

The observed reprogramming of CD8^+^ T cells may be an elegant mechanism by which the number of immune cells available for CMV control is dramatically increased. Since reprogrammed CD8^+^ T cells express Fc receptors and activating NK cell receptors, they can contribute to IgG-independent antiviral function.

Mechanistically, Semmes and colleagues showed that Fc receptor–expressing CD8^+^ T cells upregulated transcription factors highly expressed in NK cells, such as EOMES and T-bet. Moreover, these cells had lower expression of the transcription factor BCL11B, which has been observed in adult innate-like CD8^+^ T cells responding to HCMV (19). Together, the data suggest that congenital CMV infection leads to innate-like differentiation of conventional CD8^+^ T cells, which assist Fc receptor–dependent viral control (16).

## Outlook

Several interesting questions arise from the published observations (16). Is the observed innate-like differentiation in the fetus CMV-specific? Or can it be observed in congenital virus infections with other herpesviruses or even unrelated viruses such as parvovirus B19 or zikavirus (20)? Moreover, is Fc receptor expression in CD8^+^ T cells common in viral infection of adults? A recent study could not find robust Fc-γ receptor expression in circulating CD8^+^ T cells (21). However, up to 10% of lung tissue from mice infected with influenza labeled positively for Fc receptors (21), suggesting that innate programming of CD8^+^ T cells may be dependent on signals found in different tissues in adults.

Moreover, future studies should address whether NK-like CD8^+^ T cells are protective against congenital infection and/or if they are likely to alleviate CMV-dependent complications. Correlative analysis of disease outcomes with levels of Fc receptor–expressing CD8^+^ T cells could help elucidate if these cells have a protective role. If this is the case, an interesting question is whether the amount of Fc receptor–expressing cells is a limiting factor for the protective role of maternal IgG. Prior studies could not find sufficient therapeutic efficacy of IgG treatment for CMV infection during pregnancy (3). If the signals leading to innate differentiation of conventional T cells can be elucidated, boosting this path of differentiation therapeutically could be a worthwhile strategy to bolster control of congenital CMV infection.

## Version 1 01/02/2025

Electronic publication

## References

[1] Mussi-Pinhata MM (2009). Birth prevalence and natural history of congenital cytomegalovirus infection in a highly seroimmune population. Clin Infect Dis.

[2] de Vries JJ (2011). Implementing neonatal screening for congenital cytomegalovirus: addressing the deafness of policy makers. Rev Med Virol.

[3] Revello MG (2014). A randomized trial of hyperimmune globulin to prevent congenital cytomegalovirus. N Engl J Med.

[4] Leruez-Ville M (2016). In utero treatment of congenital cytomegalovirus infection with valacyclovir in a multicenter, open-label, phase II study. Am J Obstet Gynecol.

[5] Ross SA (2006). Hearing loss in children with congenital cytomegalovirus infection born to mothers with preexisting immunity. J Pediatr.

[6] Dollard SC (2007). New estimates of the prevalence of neurological and sensory sequelae and mortality associated with congenital cytomegalovirus infection. Rev Med Virol.

[7] Davison AJ (2002). Evolution of sexually transmitted and sexually transmissible human herpesviruses. Ann N Y Acad Sci.

[8] Mocarski ES (2004). Immune escape and exploitation strategies of cytomegaloviruses: impact on and imitation of the major histocompatibility system. Cell Microbiol.

[9] Dokun AO (2001). Specific and nonspecific NK cell activation during virus infection. Nat Immunol.

[10] Brown MG (2001). Vital involvement of a natural killer cell activation receptor in resistance to viral infection. Science.

[11] Hammer Q (2018). Peptide-specific recognition of human cytomegalovirus strains controls adaptive natural killer cells. Nat Immunol.

[12] Schelonka RL (1998). T cell receptor repertoire diversity and clonal expansion in human neonates. Pediatr Res.

[13] Burt TD (2013). Fetal regulatory T cells and peripheral immune tolerance in utero: implications for development and disease. Am J Reprod Immunol.

[14] Langel SN (2022). Maternal immune protection against infectious diseases. Cell Host Microbe.

[15] Vaaben AV (2022). In utero activation of natural killer cells in congenital cytomegalovirus infection. J Infect Dis.

[16] Semmes EC (2025). In utero human cytomegalovirus infection expands NK-like FcγRIII^+^CD8^+^ T cells that mediate Fc antibody functions. J Clin Invest.

[17] Pietra G (2003). HLA-E-restricted recognition of cytomegalovirus-derived peptides by human CD8+ cytolytic T lymphocytes. Proc Natl Acad Sci U S A.

[18] Clémenceau B (2008). Effector memory alphabeta T lymphocytes can express FcgammaRIIIa and mediate antibody-dependent cellular cytotoxicity. J Immunol.

[19] Sottile R (2021). Human cytomegalovirus expands a CD8^+^ T cell population with loss of BCL11B expression and gain of NK cell identity. Sci Immunol.

[20] Megli CJ, Coyne CB (2022). Infections at the maternal-fetal interface: an overview of pathogenesis and defence. Nat Rev Microbiol.

[21] Bournazos S (2020). Fc-optimized antibodies elicit CD8 immunity to viral respiratory infection. Nature.

